# Cost-utility of tDCS in depression treatment

**DOI:** 10.1192/j.eurpsy.2023.166

**Published:** 2023-07-19

**Authors:** A. Sauvaget

**Affiliations:** Psychiatry, Nantes University, Nantes, France

## Abstract

**Abstract:**

Depression is one of the most common psychiatric disorders causing considerable economic burden. However, the current treatment as usual, pharmacotherapy and psychotherapy, provides unsatisfactory treatment outcome for majority of the patients and in most cases fails to prevent treatment resistance and chronicity. tDCS, has emerged as a new neuromodulation treatment and has shown efficacy in depressed patients. To provide important insight to the payers, the cost-utility of tDCS in comparison to treatment as usual, should be clarified.

**Disclosure of Interest:**

None Declared

